# Metabolic Labeling of *Caenorhabditis elegans* Primary Embryonic Cells with Azido-Sugars as a Tool for Glycoprotein Discovery

**DOI:** 10.1371/journal.pone.0049020

**Published:** 2012-11-12

**Authors:** Amanda R. Burnham-Marusich, Casey J. Snodgrass, Anna M. Johnson, Conrad M. Kiyoshi, Sarah E. Buzby, Matt R. Gruner, Patricia M. Berninsone

**Affiliations:** Department of Biology, University of Nevada Reno, Reno, Nevada, United States of America; Johns Hopkins University, United States of America

## Abstract

Glycobiology research with C*aenorhabditis elegans* (*C. elegans*) has benefitted from the numerous genetic and cell biology tools available in this system. However, the lack of a cell line and the relative inaccessibility of *C. elegans* somatic cells *in vivo* have limited the biochemical approaches available in this model. Here we report that *C. elegans* primary embryonic cells in culture incorporate azido-sugar analogs of N-acetylgalactosamine (GalNAc) and N-acetylglucosamine (GlcNAc), and that the labeled glycoproteins can be analyzed by mass spectrometry. By using this metabolic labeling approach, we have identified a set of novel *C. elegans* glycoprotein candidates, which include several mitochondrially-annotated proteins. This observation was unexpected given that mitochondrial glycoproteins have only rarely been reported, and it suggests that glycosylation of mitochondrially-annotated proteins might occur more frequently than previously thought. Using independent experimental strategies, we validated a subset of our glycoprotein candidates. These include a mitochondrial, atypical glycoprotein (ATP synthase α-subunit), a predicted glycoprotein (aspartyl protease, ASP-4), and a protein family with established glycosylation in other species (actin). Additionally, we observed a glycosylated isoform of ATP synthase α-subunit in bovine heart tissue and a primate cell line (COS-7). Overall, our finding that *C. elegans* primary embryonic cells are amenable to metabolic labeling demonstrates that biochemical studies in *C. elegans* are feasible, which opens the door to labeling *C. elegans* cells with other radioactive or azido-substrates and should enable the identification of additional post-translationally modified targets and analysis of the genes required for their modification using *C. elegans* mutant libraries.

## Introduction

Glycosylation is a ubiquitous and important post-translational modification. More than 50% of eukaryotic proteins are glycosylated [1] and glycoproteins mediate numerous essential biological functions, including development, immune response, molecular trafficking and signal transduction [2].

Most glycosyltransferases and glycosidases exhibit a high degree of evolutionary conservation [2], which can be exploited to study glycan biosynthesis and glycoprotein function in model organisms. The nematode *C. elegans* has a strong history as a model system for human disease [3], and it is particularly advantageous for glycobiology research since numerous viable and publicly available glycosylation pathway mutants already exist. However, despite the abundance of cell biology and genetic tools available in *C. elegans*, the chemical biology approaches available in this system are much more limited [3].

**Figure 1:**
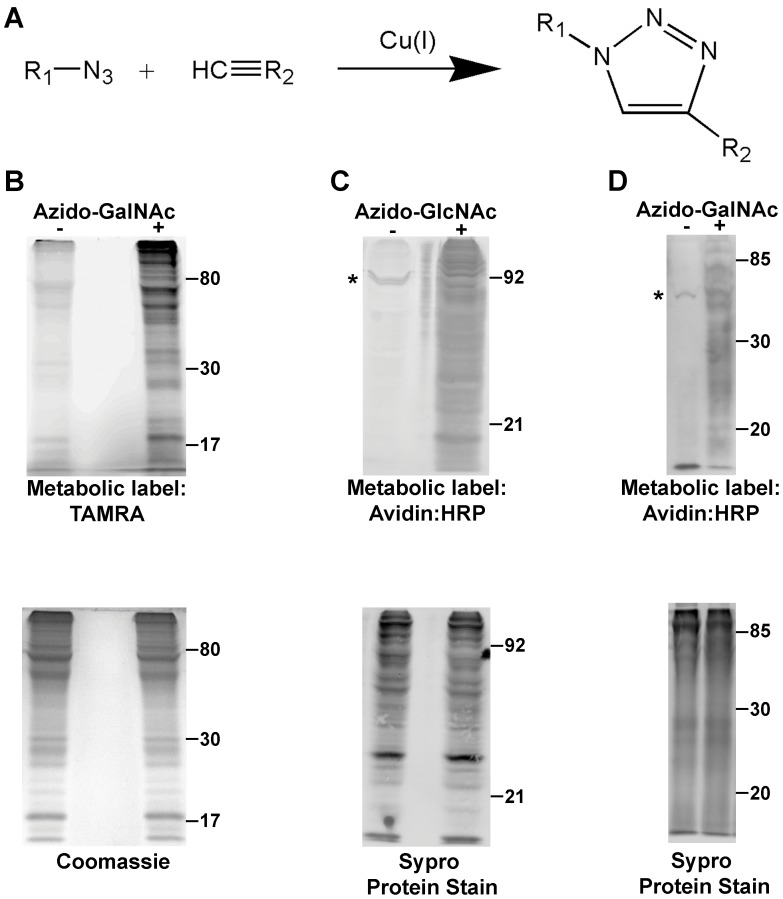
Primary *C. elegans* cells metabolically incorporate azido-GalNAc and azido-GlcNAc into cellular and secreted proteins. (A) During copper-catalyzed azide-alkyne cycloaddition (“Click Chemistry”), a covalent bond is formed between a terminal alkyne and an azide group. This reaction can be used to covalently label glycoproteins with biotin or fluorescent tags. This occurs when the R_1_ group attached to the azide is a monosaccharide linked to a glycoprotein and the R_2_ group attached to the alkyne is biotin or a fluorophore (e.g. TAMRA). (B) N2 cells were cultured with 40 µM GalNAc (−) or 40 µM azido-GalNAc (+) and cell lysates were reacted with TAMRA-alkyne by Click Chemistry. Metabolically labeled proteins were detected by TAMRA fluorescence, and protein loading was verified by Coomassie gel staining. (C) N2 cells were cultured with 40 µM GlcNAc (−) or 40 µM azido-GlcNAc (+) and cell lysates were reacted with biotin-alkyne by Click Chemistry. Metabolically labeled proteins were detected by avidin-HRP with AEC (colorimetric detection), and protein loading was verified by staining with the fluorescent protein stain, Sypro. (D) Conditioned media collected from primary embryonic N2 cell cultures grown without serum in the presence of 40 µM GalNAc (−) or 40 µM azido-GalNAc (+) was reacted with biotin-alkyne. Metabolically labeled secreted proteins were detected by avidin-HRP with ECL, and protein loading was verified by Sypro staining. Asterisk in (C) and (D) marks a known 83 kDa endogenous avidin-binding protein [34].

The purpose of our study was to expand the experimental tools available in this system by developing an approach to identify *C. elegans* glycoprotein candidates biochemically via azido-labeled sugars and Click Chemistry. Recently, a new strategy to metabolically label glycoproteins with azide-tagged analogs of natural sugars was developed [4]. The azido-labeled glycoproteins are detected by reacting the sample with labeled phosphine-probes via Staudinger ligation [4], cyclooctyne probes via strain-promoted cycloaddition [5], terminal alkyne-probes via Cu(I)-catalyzed azide-alkyne cycloaddition (i.e. Click Chemistry) [6], or difluorinated cyclooctyne (DIFO) probes via copper-free Click Chemistry [7]. All reactions generate a covalent bond specifically between the azido-analog and the labeled probe. In this study, we have used the Cu(I)-catalyzed azide-alkyne cycloaddition (Click Chemistry) reaction of a terminal alkyne-probe with an azido-labeled glycoprotein to detect metabolically labeled glycoproteins (Fig. 1A). Peracetylated azido-analogs of N-acetylglucosamine (azido-GlcNAc) have been previously used to metabolically label and identify glycoproteins in mammalian cells in culture [8]. Furthermore, peracetylated azido-analogs of N-acetylgalactosamine (azido-GalNAc) have been used to label glycoproteins and observe their spatial and temporal dynamics in adult mice [9], zebrafish embryos [10], and intact *C. elegans* nematodes [11]. However, the incorporation of azido-labeled precursors and detection reagents in intact *C. elegans* nematodes is restricted to certain cells and tissues [11], and thus it is unclear whether the incorporation efficiency of the azido-sugar *in vivo* is sufficient for downstream glycoprotein identification. Even classic metabolic labeling studies using radiolabeled substrates [Berninsone, P.M.; unpublished data] have had limited efficacy in *C. elegans,* in part due to the well-known impermeability of the nematode cuticle and the difficulty of extracting intact cells from adult animals [3]. There are no *C. elegans* cell lines, but recently a protocol was developed to isolate primary cells from *C. elegans* embryos [12]. This protocol has been used for electrophysiological and cell biological studies, but the potential of these cultures for biochemical analysis has not yet been tapped. We first optimized this cell culture system for large-scale culturing suitable for biochemical analysis and then sought to determine if *C. elegans* cells in culture are amenable to metabolic labeling with azido-sugars. Here, we present our findings that *C. elegans* primary embryonic cells in culture metabolize both azido-GalNAc and azido-GlcNAc, that the labeled glycoproteins can be detected and analyzed, and that with this method we have identified several novel *C. elegans* glycoproteins, of which an unexpectedly large proportion are mitochondrially-annotated proteins.

## Results and Discussion

### *C. elegans* Glycoproteins can be Labeled with Azido-analogs of GalNAc and GlcNAc

Large-scale primary cell cultures from dissociated N2 embryos were incubated with either GalNAc or a peracetylated azido analog of GalNAc (azido-GalNAc), while parallel cultures were incubated with either GlcNAc or a peracetylated azido analog of GlcNAc (azido-GlcNAc). To detect the azido-label, cell lysates were reacted via Click Chemistry with a fluorescently-conjugated alkyne probe (tetramethylrhodamine (TAMRA)-alkyne), and the labeled proteins were visualized directly by in-gel fluorescence (Fig. 1B). Additionally, the azido-label could also be detected by Click Chemistry reaction with a biotin-alkyne probe and subsequent visualization using avidin-HRP (Figure 1C). Both azido-sugars were metabolically incorporated as shown by robust TAMRA or avidin-HRP signals in the azido-sugar lysate compared to the control lysates (Fig1B and C). Furthermore, secreted *C. elegans* proteins collected from the conditioned media of cell cultures grown without serum in the presence of GalNAc or azido-GalNAc could also be metabolically labeled and detected (Fig. 1D). Importantly, the detection of azido-specific signal was dependent on the Click Chemistry reaction with the alkyne-probe, since azido-GalNAc lysates showed an increase in alkyne-probe signal relative to GalNAc lysates only after Click Chemistry reaction (Fig. S1). Additionally, azido-GalNAc metabolic labeling did not prevent differentiation of the primary cell cultures, since cultures of the NW1229 strain, which contains the post-mitotic, pan-neuronal marker F25B3.3::GFP, still expressed GFP (data not shown).

Recently, using a complementary experimental approach, Laughlin and Bertozzi reported the incorporation of azido-GalNAc but not azido-GlcNAc *in vivo* into *C. elegans* glycoproteins [11]. This difference may be due to the different azido-GlcNAc labeling and detection strategies used in their study. Laughlin *et al*.’s use of whole worms with an intact cuticle enabled the observation of the tissue localization of the labeled glycoproteins, but may have presented a permeability challenge to the azido-sugar or the fluorescent-probe, which would account for both the undetectable azido-GlcNAc labeling and their observation that azido-GalNAc predominantly labeled organs exposed to the external environment (*i.e.* vulva, pharynx).

We next investigated the glycoprotein profile of azido-GalNAc metabolic incorporation. The *ogt-1* mutant line has a simplified glycoprotein profile due to a lack of O-GlcNAc glycoproteins caused by mutation of the single *C. elegans* glycosyltranferase responsible for O-GlcNAc addition [13]. Lysates from *ogt-1* cells incubated with azido-GalNAc were 1) treated with the endoglycosidase pNGaseF to cleave N-linked glycans, 2) mock-treated or 3) left untreated. Although the expected SDS-PAGE mobility shift was observed in a pNGaseF digestion run in parallel on the N-glycosylated, positive control protein (RNaseB), which indicates that the enzyme was active, the *ogt-1* lysates that were pNGaseF treated, mock-treated or left untreated all displayed comparable azido-labeling (Fig. S3). These results indicate that the majority of the azido-GalNAc label is incorporated into glycan classes that are insensitive to pNGaseF, which is consistent with observations of intact *C. elegans* worms labeled with azido-GalNAc *in vivo* [11].

### Azido-metabolic Labeling of *C. elegans* Cells Generates Glycoprotein Candidates

To determine the utility of azido-labeling for identifying *C. elegans* glycoproteins, lysates from N2 cells incubated with azido-GalNAc or GalNAc were reacted with TAMRA-alkyne and analyzed by 2D gel electrophoresis (2DE). Selection of 2DE glycoprotein candidate spots for mass spectrometry (MALDI-TOF/TOF) identification was based on a three-tiered, stringent decision process. First, in two independent experiments, a spot had to exhibit TAMRA fluorescence only in the azido-GalNAc gel or >3-fold higher TAMRA fluorescence in the azido-GalNAc gel than in the GalNAc gel. Second, the spot had to have enough protein (as visualized by Sypro staining) to have a reasonable chance of identification by mass spectrometry. Therefore, spots that met the first criteria but had too little protein were not analyzed by mass spectrometry. Third, once the mass spectrometry analysis had been performed as described in Methods, a protein identification was considered valid only if supported by a significant MASCOT score (MASCOT scores >82; MASCOT expect p-value <0.05) and more than 5 unique peptides (Table S1 and Dataset S1). Lastly, it is important to note that mass spectrometry was not used to determine if a protein was glycosylated; it was used to identify proteins whose glycosylation status had already been indicated by previous experiments (*i.e.* azido-sugar metabolic labeling, lectin affinity purification, galactosyltransferase labeling, etc.).

These stringent criteria generated multiple glycoprotein candidates, an unexpectedly large proportion of which were mitochondrially-annotated proteins (9 of 20 candidates; Table S1). Historically, reports of glycosylated isoforms of mitochondrial proteins have been limited to rare, isolated accounts [14], [15]. But recently, several high-throughput proteomics experiments have identified glycosylated isoforms of multiple mitochondrially-annotated proteins [16], [17]. Our observation that mitochondrially-annotated proteins comprise 45% of our *C. elegans* glycoprotein candidates is much higher than the proportion recently reported in a high-throughput screen in yeast (6%; 30/534) [17]. Beyond the difference in species, this difference likely reflects a bias of the metabolic labeling with 2D gel and mass spectrometry approach for high-abundance glycoproteins. We suggest that although glycosylated isoforms of mitochondrially-annotated proteins do not likely comprise 45% of all cellular glycoproteins, our detection of nine such glycoproteins in a single experiment does suggest that glycosylated isoforms of mitochondrially-annotated proteins may occur more frequently than the sparse literature has previously indicated. Moreover, our detection of multiple mitochondrially-annotated proteins as glycoprotein candidates suggests several significant questions regarding the cell biology of how such proteins become glycosylated, whether the glycosylated isoforms actually reside in the mitochondria, and what the functional significance of such glycosylated isoforms is.

**Figure 2:**
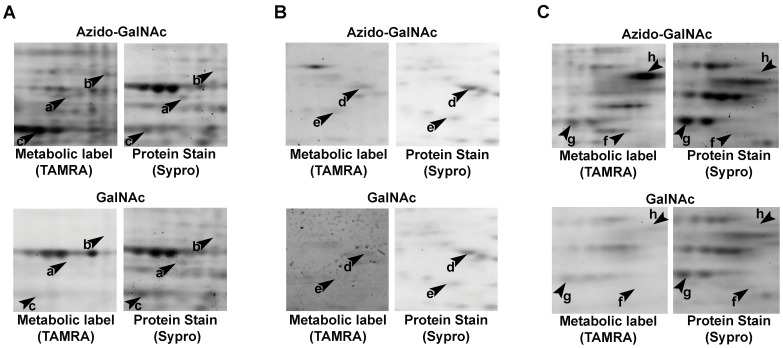
Azido-metabolic labeling of *C. elegans* cells generates glycoprotein candidates. *C. elegans* embryonic cells grown with 40 µM azido-GalNAc (top panels) or 40 µM GalNAc (bottom panels) were reacted with TAMRA-alkyne and fractionated by 2DE. (A), (B), and (C) each show a different field of view representative of two independent experiments. Within each field of view, metabolically labeled glycoproteins were detected in-gel by their TAMRA fluorescence (left panels). And subsequently, the total protein profile of the sample was visualized with Sypro protein stain (right panels). Arrowheads mark glycoprotein candidates that met the criteria listed in Results and Discussion. Some spots marked with arrowheads were cut from the gels shown while others were cut from the gels of the other independent experiment. MALDI-TOF/TOF identified the following *C. elegans* proteins within each arrowhead-marked spot: (a) ATP synthase α-subunit (H28O16.1); (b) Acyl CoA Dehydrogenase family member (*acdh-12*); (c) Ubiquinol-Cytochrome c oxidoreductase complex family member (*ucr-2.3*); (d) Actin (peptides shared by ACT-2, ACT-3, and ACT-4 were observed); (e) TCTP (translationally-controlled tumor protein) homolog family member (*tct-1*); (f) Aspartyl protease family member (*asp-4*); (g) Tubulin, Beta family member (*tbb-1*); (h) Tubulin, Beta family member (*tbb-1*). TBB-1 was found in two different 2DE spots likely due to post-translational modification or proteolytic processing.

The major caveat of 2DE is the possibility that multiple proteins in a sample may have similar enough molecular weights and isoelectric points that they all migrate to the same place on the gel, thereby creating a protein spot in which only the most abundant protein is identified by mass spectrometry. Thus we consider the proteins identified from our selected TAMRA-positive 2DE protein spots to be *glycoprotein candidates*, and *identified glycoproteins* to be those whose glycosylation is verified by at least one additional, independent experimental approach. To determine if our method is indeed a useful tool for facilitating glycoprotein discovery in *C. elegans,* we tested the glycosylation status of several of our candidates using independent experimental approaches. We chose to focus on the following particularly interesting protein spots (Fig. 2, arrowheads a, d, and f), which were identified by MALDI-TOF/TOF as: ATP synthase α-subunit (H28O16.1), actin, and aspartyl protease-4 (ASP-4), respectively (Table S1 and Dataset S1). Together, these glycoprotein candidates comprise examples of a mitochondrial, atypical glycoprotein, a protein family whose glycosylation has been reported in other species, and a novel but predicted glycoprotein.

### The Mitochondrial Protein ATP Synthase α-subunit has a Glycosylated Isoform

**Figure 3:**
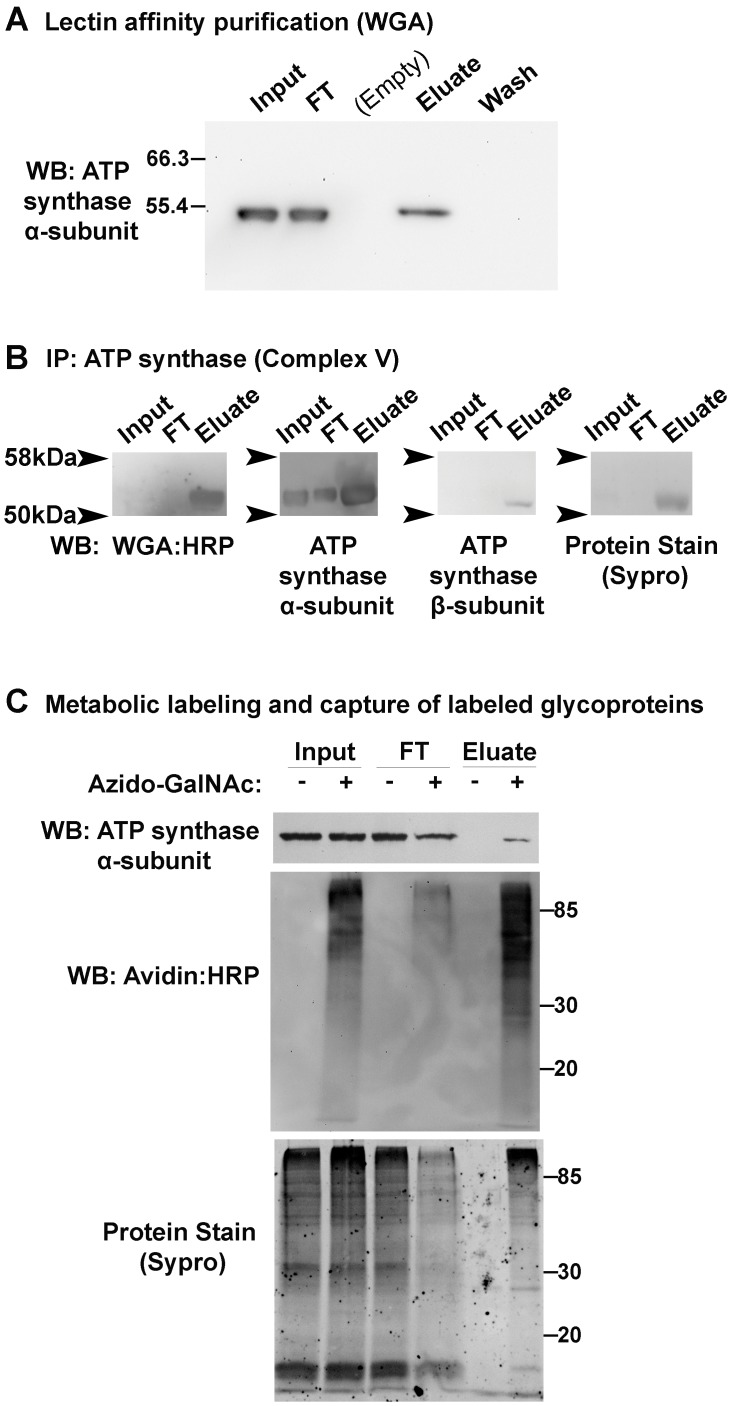
ATP synthase α-subunit has a glycosylated isoform. (A) Glycoproteins were purified from bovine heart extracts enriched for mitochondria by incubating the detergent-solubilized proteins with Wheat Germ Agglutinin (WGA) agarose beads. After extensive PBS washing, the captured glycoproteins were specifically eluted with PBS supplemented with 0.5 M GlcNAc, which is a monosaccharide that competes with glycoproteins for the sugar-binding sites on the WGA-beads. Input and non-binding flowthrough (FT) lanes contain 0.03% of each fraction. The entirety of the last wash and the eluate fractions and were precipitated, resuspended in Laemmli buffer, and loaded into their respective gel lanes. ATP synthase α-subunit was detected by western blot. (B) Bovine heart extracts enriched for mitochondria were immunoprecipitated with an ATP synthase Complex V capture antibody. Input, FT, and eluate fractions were loaded in duplicate lanes. One half of the blot was probed with WGA:HRP, then stripped and probed with ATP synthase β-subunit antibody; the other half of the blot was probed with ATP synthase α-subunit antibody. Representative Sypro staining from the WGA-probed blot half is shown. (C) COS-7 cells were incubated with 50 µM azido-GalNAc (+) or 50 µM GalNAc (−). Lysates were reacted with biotin-alkyne, and biotinylated azido-labeled proteins were captured with streptavidin beads. Input and FT lanes contain 2.5% of sample; eluate lanes, 50%. Azido-GalNAc and GalNAc samples were always handled in parallel.

Despite the prevalence of glycosylation in the proteome, few mitochondrial glycoproteins have been identified. Identification of ATP synthase α-subunit as a glycoprotein candidate (Fig. 2A, Table S1, and Dataset S1) illustrates the capability of our method to identify unexpected and atypical glycoproteins. To verify the glycosylation of ATP synthase α-subunit, two independent approaches were pursued. In the first, glycoproteins were affinity purified from enriched bovine heart mitochondria and N2 larval lysates using beads conjugated to the lectin Wheat Germ Agglutinin (WGA). The beads were washed extensively in wash buffer, and then captured glycoproteins were specifically eluted by incubating in wash buffer supplemented with the competing sugar, GlcNAc. Western blotting of the bovine samples revealed that ATP synthase α-subunit was not released during the last wash, but was released when the beads were subsequently incubated in wash buffer with GlcNAc (Fig. 3A). *C. elegans* ATP synthase α-subunit was also detected by Western blot in the fraction specifically eluted with GlcNAc (Fig. S2). In a reciprocal experiment, bovine heart extract enriched for mitochondria was immunoprecipitated with an ATP synthase capture antibody. The ATP synthase complex was pulled down, including a protein band reactive with both an ATP synthase α-subunit antibody and the lectin WGA (Fig. 3B). WGA-binding specificity was demonstrated by the reduction in WGA signal when the blot was probed with WGA and 0.5 M GlcNAc (data not shown). To address whether the glycosylation of the α-subunit of ATP synthase is further evolutionarily conserved, COS-7 (*Cercopithecus aethiops*) cells were incubated with azido-GalNAc or GalNAc and equal amounts of biotin-alkyne reacted proteins were subjected to streptavidin enrichment. Western blotting showed that ATP synthase α-subunit was eluted only from the azido-GalNAc lysate, indicating that the α-subunit is glycosylated in nematodes (*C. elegans*), mammals, (*Bos taurus*), and a primate cell line (*C. aethiops*) (Fig. 3C). Previously, ATP synthase α-subunit was identified as one of many glycoprotein candidates in a genome-wide, lectin binding assay in yeast [17] and more recently, as either a binding partner of a glycoprotein or a glycoprotein itself in a high-throughput immunoprecipitation experiment using an O-GlcNAc-specific antibody in *C. elegans* [18]. However, false positives are a risk in high-throughput experiments, and in neither of these high-throughput experiments was the glycosylation of ATP synthase α-subunit confirmed by an independent, individualized approach. Our data extend these observations, validate the glycosylation of ATP synthase α-subunit, and indicate that its glycosylation is evolutionarily conserved in nematode, bovine, and primate species. Furthermore, our detection of glycosylated ATP synthase α-subunit demonstrates that azido-labeling *C. elegans* primary embryonic cells can generate atypical *C. elegans* glycoprotein candidates.

The previous genome-wide, lectin binding study in yeast did not assess the proportion of glycosylated ATP synthase α-subunit relative to the total [17]. In our experiments, only a fraction of ATP synthase α-subunit bound the WGA-beads (Fig. 3A and S2), and azido-labeling with biotin-alkyne reaction and streptavidin purification captured some but not all ATP synthase α-subunit (Fig. 3C). The presence of unbound ATP synthase α-subunit in the flowthrough lane of Figs. 3A and 3C was observed despite the use of a large amount of WGA- and streptavidin-beads that was unlikely to have been limiting, as detailed in Materials and Methods. Furthermore, the amount of glycosylated ATP synthase α-subunit captured during each of these experiments is similar to the amount of ATP synthase α-subunit present in the input lanes of each experiment. Because the eluate lanes contain all glycoproteins captured during the experiments yet the input lanes contain only 0.03% or 2.5% of the starting material in the WGA affinity or streptavidin capture experiment, respectively, this indicates that the glycosylated isoform of ATP synthase α-subunit is a relatively low abundance isoform.

In addition, our detection of glycosylated ATP synthase α-subunit in the enriched mitochondria fraction (Fig. 3A and 3B) does not necessarily imply that this isoform is localized within the mitochondria. Interestingly, ATP synthase α-subunit can localize to the plasma membrane in multiple cell types [19], although its function at the membrane is unknown. Moreover, glycosylated ATP synthase α-subunit has been observed at the surface of murine neurons [20]. Whether glycosylation is functionally important for localizing some of the expressed protein to the plasma membrane remains to be tested. Alternatively, glycosylation of the α-subunit may affect ATP synthase activity, since increased O-GlcNAcylation of Complex I, III, and IV subunits is associated with impaired respiratory activity in rat cardiac myocytes [21].

### Actin has a Glycosylated Isoform in *C. elegans*

**Figure 4:**
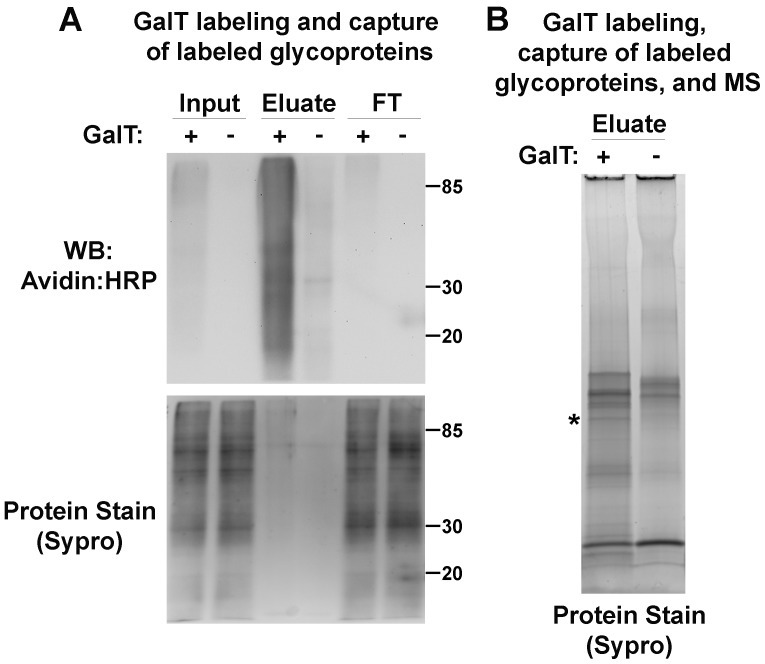
Actin has a glycosylated isoform in *C. elegans*. (A) Glycoproteins containing terminal GlcNAc were purified by first treating N2 larval lysates with both UDP-azido-GalNAc and GalT (+), or with UDP-azido-GalNAc alone (−), then reacting with biotin-alkyne, and finally capturing the biotinylated glycoproteins with streptavidin beads. Biotinylated glycoproteins were visualized by Western blot with avidin:HRP. (B) Eluted glycoproteins were purified as in (A), fractionated by SDS-PAGE, and the gel was stained with Sypro. Peptides shared by ACT-2, ACT-3, and ACT-4 were identified by MALDI-TOF/TOF from the asterisk-marked band, which was exclusive to the (+) lane.

The glycosylation of actin family members is well-established in mammalian systems, and the glycosylated sites of rat alpha cardiac actin were recently mapped [22]. The identified sites are conserved in *C. elegans* ACT-2, ACT-3, and ACT-4. After identifying actin as a *C. elegans* glycoprotein candidate by azido-metabolic labeling and MALDI-TOF/TOF (Fig. 2B, Table S1, and Dataset S1), we confirmed its glycosylation by treating or mock treating *C. elegans* larval lysates with a recombinant mutant β-1, 4-galactosyltransferase (GalT) and UDP-azido-GalNAc to enzymatically label terminal GlcNAc-containing glycoproteins. Labeled glycoproteins were then reacted with biotin-alkyne and purified with streptavidin-beads (Fig. 4A). MALDI-TOF/TOF identified actin within a protein band exclusive to the GalT treated eluate (Fig. 4B; Table S2 and Dataset S2). To confirm that actin is glycosylated in *C. elegans*, two independent experiments were performed (Figure S4). In panel A, lysates of adult *C. elegans* were labeled with azido-GalNAc via mutant GalT or mock labeled (no GalT). After reacting the azido-tagged glycoproteins with biotin-alkyne via Click Chemistry, the labeled glycoproteins were purified using streptavidin beads, and analyzed by Western blot using an anti-actin antibody. The anti-actin antibody reacted with a glycoprotein band captured only from the GalT treated sample and not from the mock GalT treated sample. Similarly, in S4B, lysates of adult *C. elegans* were applied to WGA beads, washed extensively and the captured proteins eluted with the competing sugar (GlcNAc). A Western blot using an anti-actin antibody showed that a fraction of the actin protein is present in the eluted fraction but not in the last wash. Together, these results strongly suggest that a fraction of *C. elegans* actin is indeed glycosylated.

The complete sequences of *C. elegans* ACT-2, ACT-3, and ACT-4 differ only at two amino acids (UniProtKB/Swiss-Prot accession numbers P10984, P10983, P10986, respectively), and the peptides observed do not distinguish between these proteins (data not shown). Thus it is possible that more than one of these actin family members may be glycosylated in *C. elegans*. Indeed, peptides shared by ACT-1, ACT-2, ACT-3, and ACT-4 were identified by a high-throughput O-GlcNAc antibody immunoprecipitation experiment as either candidate O-GlcNAcylated proteins or binding partners of such proteins [18].

Interestingly, in mammalian systems, actin is glycosylated with O-linked GlcNAc [22]. O-GlcNAc modified glycoproteins contain a single GlcNAc residue attached to a serine or threonine. Thus our detection of glycosylated actin via azido-GalNAc metabolic labeling suggests that UDP-azido-GalNAc may epimerize to UDP-azido-GlcNAc within *C. elegans* primary embryonic cells, which is consistent with similar observations in mammalian cell lines [23]. Interconversion is also supported by our observation that several other of our *C. elegans* glycoprotein candidates generated by azido-GalNAc labeling are nucleocytosolic proteins with reported O-GlcNAc glycosylation [8] (Table S1). However, the comparable azido-signal of lysates from azido-GalNAc treated wildtype and *ogt-1* cells, which lack the O-GlcNAc transferase, (Fig. S5) suggests that many glycoproteins other than O-GlcNAc glycoproteins comprise the bulk of the detectable azido-label.

### The Predicted Glycoprotein ASP-4 is Glycosylated

The *C. elegans* aspartyl protease ASP-4 is required for necrotic neurodegeneration mediated by increased intracellular calcium [24], and it contains a putative N-glycosylation site at amino acid 106 [25]. However, the glycosylation of ASP-4 has not been directly tested. As an additional validation of the utility of our approach for identifying novel but predicted glycoproteins, we verified our detection of ASP-4 as an azido-labeled glycoprotein candidate (Fig. 2C; Table S1, and Dataset S1) by an independent approach: lectin affinity purification with 2DE fractionation, ProQ Emerald Glycoprotein staining, and MALDI-TOF/TOF identification (Fig. S6A and B, Table S2, and Dataset S2). Lack of antibodies against ASP-4 or a transgenic strain expressing a translational ASP4::GFP fusion preclude additional verification.

In summary, our findings show that glycoproteins in *C. elegans* primary embryonic cells can be metabolically labeled with azido-sugars, detected with proteomics-compatible alkyne probes, and identified by MALDI-TOF/TOF. The utility of this method for enhancing our understanding of the cell biology of glycosylation has already been demonstrated by our surprising detection of multiple mitochondrially-annotated proteins as atypical glycoprotein candidates whose glycosylation has not been previously reported in *C. elegans*. This observation is biologically significant because it suggests that glycosylation of mitochondrially-annotated proteins may occur more frequently than was previously thought, thereby opening the door to a number of future questions. In experiments designed to elaborate on this observation, we recently determined that the glycosylation extends beyond mitochondrially-annotated proteins: multiple proteins with established function in the mitochondria have low-abundance glycosylated isoforms in bovine heart *in vivo* [26].

Furthermore, azido-labeling of primary *C. elegans* embryonic cells should facilitate future downstream glycoproteomic analyses in *C. elegans*, including identification of additional novel glycoproteins and the mapping of their glycosylation sites. In addition, fluorescent alkyne probes could be used for 2DE analysis of differentially charged glycoprotein isoforms between *C. elegans* mutants.

Additionally, a major challenge in glycobiology has been the identification of glycosyltransferase targets, as each individual glycosyltransferase can modify many protein targets. The application of azido-sugar metabolic labeling to a genetically tractable model organism with multiple glycosyltransferase mutants already available should thus enable the rapid discovery of glycosyltransferase targets through comparative glycoproteomic analyses of the mutants. Our approach could also be used to identify or compare the glycosylation of specific targets in different *C. elegans* cell types (*e.g.* muscle *vs.* neuron) by incorporating a published method for fluorescence-activated cell sorting [27] on the cultured cells. Similarly, our approach could also be used to compare the glycosylation of specific targets in different *C. elegans* life stages using a published protocol for culturing *C. elegans* larval cells [28].

Beyond glycomics, the amenability of *C. elegans* primary embryonic cells to metabolic labeling demonstrates the feasibility of biochemical studies in *C. elegans*, and also suggests that metabolic labeling of *C. elegans* mutant libraries with classical radioactive isotopes or other classes of azido-analogs (*e.g.* azido-farnesyl) [29], ω-azido fatty acids (N-myristoylation and S-acylation) [30], may facilitate the future detection and functional analysis of other post-translationally modified proteins and post-translational modification pathway components.

## Materials and Methods

### *C. elegans* Maintenance and Strains Used

The wild-type (N2) and ogt-1*(ok430)* mutant strains were obtained from the CGC and maintained as described [31].

### Cell Culture Generation and Metabolic Labeling

Primary embryonic *C. elegans* cells were generated as described [12] with these modifications: large quantities of pseudo-synchronized gravid hermaphrodites were produced by starving worms and starting 10–40 individual 600 cm^2^ plates with arrested L1 larvae; chitinase (Sigma) digestion was performed for 1.25 hrs in a maximal volume of 15 ml per 50 ml tube; embryos were dissociated with a 3 ml syringe and a 26G 5/8′′ needle; cells were cultured in L15 media (Invitrogen) supplemented with 50 U/ml penicillin, 50 µg/ml streptomycin with 10% fetal bovine serum (FBS, Invitrogen); and cells were plated at a density of 1.3×10^6^ cells/cm^2^. *C. elegans* cells were cultured for 20–72 hrs in media containing 40 µM sugar or peracetylated azido-sugar (Invitrogen); COS-7 cells (from ATCC) were cultured for 48 hrs in 50 µM sugar or peracetylated azido-sugar. Cells were harvested by incubating 30 min on ice in 1% SDS 50 mM TrisHCl, pH 8 with 1X protease inhibitor cocktail (CalBiochem) and 300 U/ml Benzonase (Novagen). Azido-GlcNAc- and GlcNAc-treated cells were incubated in 4 mM GlcNAc for 1 hr prior to harvest and were washed with PBS +4 mM GlcNAc to inhibit O-GlcNAcase activity.

### Detection of Metabolically Labeled Proteins

40–200 µg of protein in 50 µl of lysis buffer were reacted with final concentrations of 20 µM biotin- or TAMRA-alkyne, 47.5% DMSO, 2 mM fresh ascorbic acid, 200 µM Tris[(1-benzyl-1H-1,2,3-triazol-4-yl)methyl]amine, and 2 mM CuSO_4_ in a 200 µl reaction volume. Click Chemistry reactions were incubated for 1 hr at room temperature (RT), then were precipitated with methanol-chloroform.

### Western Blot Analysis

To detect azido-proteins reacted with biotin-alkyne or WGA-reactive glycoproteins, membranes were incubated 1 hr with 0.01 µg/ml avidin:HRP or 0.5 µg/ml WGA:HRP respectively (Vector Labs). To detect ATP synthase α- and β-subunits, blots were probed with anti-ATP synthase α- or β-subunit (MitoSciences MS507 or MS503, respectively) at 1∶1000 at 4°C overnight and developed as per company recommendations. The goat anti-mouse IgG:HRP secondary antibody (Jackson ImmunoResearch, 115-035-062) was used at 1∶10,000.

### Lectin Affinity Purification of Bovine Heart Extracts

The basic mitochondria enrichment protocol of [32] was modified as follows: 1) a polytron was used to homogenize 400 ml batches of bovine heart (20 s at 14,000 RPM); 2) nuclei and tissue clumps were removed by a 10 min 1,000×*g* centrifugation instead of 20 min at 1,200×*g*; 3) three 15 min centrifugations at 12,000×*g* instead of 26,000×*g* were used to pellet and wash the mitochondria. Mitochondria at 1.8 mg/ml were solubilized in detergent buffer (0.5 mM PMSF, 3% w/v CHAPS, 7M urea, 2M thiourea, 9 mM Tris-acetate, pH 7.0) on ice for 30 min. After a 30 min centrifugation at 16,000×*g,* 5 mg of supernatant protein was incubated for 1 hr at RT with 0.3 ml WGA conjugated agarose beads (Vector). This is sufficient WGA-beads to capture 2.4 mg of glycoproteins carrying terminal GlcNAc or sialic acid residues. 2.4 mg would comprise half of the total sample applied to the beads, which is an unlikely large amount given that only a fraction of cellular glycoproteins are modified with terminal GlcNAc or sialic acid. After six 2.5 min washes in 2 ml PBS, WGA-beads were incubated 2X 10 min in 0.3 ml PBS supplemented with 0.5 M GlcNAc to specifically elute captured glycoproteins.

### Streptavidin Enrichment

In parallel, 2 mg each of azido-GalNAc and GalNAc COS-7 cell lysates were reacted with biotin-alkyne and biotinylated azido-labeled proteins were enriched with excess streptavidin beads (GE) as described [8]. Streptavidin beads were considered to be in excess, because although not every protein in the 2 mg of cell lysate is a glycoprotein, the beads had the capacity to bind 2 mg of glycosylated proteins.

### 2D Gel Electrophoresis (2DE) and Analysis

Samples were isoelectrically focused as per [33] with modifications described in detail in Supplementary Methods and References S1. After electrophoresis, TAMRA fluorescence was recorded with a Typhoon Trio (GE) (ex 532 nm; em 580 nm BP). Gels were then stained with Sypro Ruby (Invitrogen), and imaged with a Typhoon Trio (ex 488 nm, em 610 nm BP) or Bio-Rad VersaDoc 4000 imager (ex 300 nm, em 520 nm LP). Gels were analyzed using Bio-Rad PDQuest 8.0, and spots excised using Bio-Rad ExQuest Spot Cutter. See Supplementary Methods and References S1 for details on MALDI-TOF/TOF methods used for Supplementary Tables. Only protein identifications that had significant MASCOT scores (MASCOT scores >82; MASCOT expect p-value <0.05) and ≥5 unique peptides were accepted as valid.

### ATP Synthase Complex V Immunocapture

Capture was performed on mitochondria and membrane enriched fraction from bovine heart (MitoSciences MS802) with ATP Synthase Complex V Capture antibody (MitoSciences MS501) as per manufacturer recommendations.

### GalT Labeling and Enrichment

N2 L1/L2 samples were sonicated in PBS and protease inhibitors (CalBiochem) 10 min, then centrifuged 1 hr at 16,000×*g*. The cleared supernatant was then labeled with (or mock-labeled without) mutant GalT and UDP-azido-GalNAc (Invitrogen), as per manufacturer’s instructions. Samples were then reacted with biotin-alkyne and subjected to streptavidin-enrichment as above.

## Supporting Information

Figure S1 **Detection of azido-GalNAc-specific avidin:HRP signal in** ***C. elegans*** **cell lysates is dependent on Click Chemistry reaction of lysates with biotin-alkyne and copper catalyst.** Primary embryonic N2 *C. elegans* cells were metabolically labeled with GalNAc or azido- GalNAc for 24 hrs, then the metabolic label was detected by reacting cell lysates in the described combinations of biotin-alkyne and copper catalyst. Reactions containing a constant amount of copper and various dilutions of biotin-alkyne were also tested.
(PDF)

Figure S2 **A WGA-binding glycosylated isoform of ATP synthase α-subunit is present in** ***C. elegans*** **.** *C. elegans* glycoproteins were solubilized in detergent buffer and purified using WGA-agarose. ATP synthase α-subunit was present in both the non-binding flowthrough (FT) fraction and the fraction specifically eluted with GlcNAc.
(PDF)

Figure S3 **The majority of azido-signal from azido-GalNAc treated cells is incorporated into pNGaseF-insensitive glycoproteins.** *C. elegans ogt-1* cells were incubated with azido-GalNAc, and the lysates were treated with the N-glycan endoglycosidase pNGaseF, mock treated, or left untreated. The remaining azido-labeled glycoproteins were then visualized by Click Chemistry reaction with biotin-alkyne and Western blotting with avidin:HRP. Protein loading was verified by Sypro Ruby total protein staining. The N-glycosylated protein, RNaseB, was run as a positive control for pNGaseF activity. Asterisk indicates the expected molecular weight of pNGaseF.
(PDF)

Figure S4 **A portion of** ***C. elegans*** **actin is glycosylated.** (A) 450 ug of *C. elegans* adult lysates were labeled with azido-GalNAc via a mutant β-1, 4-galactosyltransferase (GalT) or mock labeled (no GalT). After reacting the azido-tagged glycoproteins with biotin-alkyne via Click Chemistry, the labeled glycoproteins were purified using streptavidin beads and analyzed by Western blot using an anti-actin antibody (Sigma A4700). Protein loading was visualized by Sypro Ruby total protein staining. Input and flowthrough (FT) lanes contain 20 ug aliquots of each fraction; eluate lane contains the entire fraction. (B) Ten milligrams of *C. elegans* adult lysates were applied to WGA beads, the beads were washed extensively with PBS, and then the captured proteins were eluted with PBS supplemented with the competing sugar (GlcNAc). Samples were analyzed by Western blot using an anti-actin antibody (Abcam ab3280). Protein loading was visualized by Sypro Ruby total protein staining. Input and FT lanes contain 20 ug aliquots of each fraction; last wash and eluate lanes contain 100% of each fraction.
(PDF)

Figure S5 **The azido-labeled protein profile of N2 lysates is comparable to that of** ***ogt-1*** **lysates.** N2 and *ogt-1* cells were incubated with 40 µM azido-GalNAc for 72 hrs, then reacted via Click Chemistry with TAMRA-alkyne, fractionated by SDS-PAGE, and electrotransferred. TAMRA signal was detected by UV on the blot, then the blot was stained with Sypro Ruby total protein stain to verify protein loading.
(PDF)

Figure S6 **ASP-4 is glycosylated.** *C. elegans* glycoproteins were purified using WGA-agarose. The fraction eluted with GlcNAc was fractionated by 2DE and analyzed with Pro-Q Emerald Total Glycoprotein Stain (A) and then with Sypro total protein stain (B). Arrowheads mark the spot identified by mass spectrometry as ASP-4.
(PDF)

Supplementary Methods and References S1 (PDF)

Table S1 ***C. elegans*** **glycoproteins candidates detected by metabolic labeling of primary embryonic cells with azido-GalNAc.** *C. elegans* cells were labeled with azido-GalNAc, the lysates reacted with TAMRA-alkyne via Click Chemistry, and the proteins separated by 2DE. The 2DE glycoprotein candidate spots selected for MALDI-TOF/TOF identification were those that in two independent experiments exhibited TAMRA fluorescence only in the azido-GalNAc gel or >3-fold higher TAMRA fluorescence in the azido-GalNAc gel *vs.* the GalNAc gel. A protein identification was considered valid only if it was supported by a significant MASCOT score (MASCOT score >82; MASCOT expect p-value <0.05) and more than 5 unique peptides. For those 2DE spots with valid protein identifications, the top-scoring MASCOT identification from a search of the NCBI nr database is listed for each 2DE spot.
(PDF)

Table S2 **MALDI-TOF/TOF identifications of candidate glycoproteins from 2DE protein spots or SDS-PAGE gel bands.** WGA affinity purification: *C. elegans* larval proteins were captured with WGA-agarose, eluted with GlcNAc, and separated by 2DE. A spot that fluoresced with Pro-Q Emerald Total Glycoprotein Stain of the appropriate molecular weight and isoelectric point to be ASP-4 was selected for MALDI-TOF/TOF identification. GalT labeling: *C. elegans* L1/L2 lysates treated or mock treated with GalT and UDP-azido-GalNAc were reacted with biotin-alkyne. Biotinylated azido-glycoproteins were then captured with avidin beads. One SDS-PAGE protein band captured specifically from GalT-treated lysates and not mock-treated lysates was selected for MALDI-TOF/TOF identification. The top-scoring MASCOT identification from a search of the NCBI nr database or, where stated, the *C. elegans* database, is listed for each 2DE spot or SDS-PAGE gel band.
(PDF)

Dataset S1 **Mass spectrometry data supporting the identification of the** ***C. elegans*** **glycoproteins candidates.** 2DE spots of metabolically labeled *C. elegans* samples matching the criteria given in the text for glycoprotein candidates were analyzed by MALDI-TOF/TOF. MALDI-TOF/TOF methods are described in detail in Supplementary Methods and References S1. Dataset S1 includes the following information for each protein identification: protein accession number, MASCOT score and expect value, sequence coverage, peptides observed, ion score if MS/MS was performed on the peptide, and the MS spectra (m/z *vs.* intensity).
(RAR)

Dataset S2 **Mass spectrometry data from glycoprotein verification experiments supporting the identification of** ***C. elegans*** **ASP-4 and actin.** ASP-4: *C. elegans* glycoproteins were purified using WGA-agarose. The fraction eluted with GlcNAc was fractionated by 2DE. Protein spots that stained positive with Pro-Q Emerald Total Glycoprotein Stain were analyzed by MALDI-TOF/TOF, and the mass spectrometry data pertaining to the spot identified as ASP-4 is shown. Actin: Glycoproteins containing terminal GlcNAc were purified by treating N2 larval lysates with both UDP-azido-GalNAc and GalT (+), or with UDP-azido-GalNAc alone (−),reacting with biotin-alkyne, and capturing the biotinylated glycoproteins with streptavidin beads. Eluted glycoproteins were fractionated by SDS-PAGE, and the gel was stained with Sypro. A protein band unique to the GalT treated sample was identified by MALDI-TOF/TOF as ACT-4; although the peptides observed are shared by *C. elegans* ACT-2, ACT-3, and ACT-4. Dataset S2 includes the following information in support of the identification of ASP-4 and actin: protein accession number, MASCOT score and expect value, sequence coverage, peptides observed, ion score if MS/MS was performed on the peptide, and the MS spectra (m/z *vs.* intensity).
(PDF)
