# Supplementary material for: Metabolic Labeling of Caenorhabditis elegans Primary Embryonic Cells with Azido-Sugars as a Tool for Glycoprotein Discovery
Source: PLoS One. 2012 Nov 12;7(11):e49020. doi: 10.1371/journal.pone.0049020 (PMC3495777; doi:10.1371/journal.pone.0049020)
Supplement: Figure S1 — Detection of azido-GalNAc-specific avidin:HRP signal in C. elegans cell lysates is dependent on Click Chemistry reaction of lysates with biotin-alkyne and copper catalyst. Primary embryonic N2 C. elegans cells were metabolically labeled with GalNAc or azido- GalNAc for 24 hrs, then the metabolic label was detected by reacting cell lysates in the described combinations of biotin-alkyne and copper catalyst. Reactions containing a constant amount of copper and various dilutions of biotin-alkyne were also tested. (PDF) [file pone.0049020.s001.pdf]

Figure S1

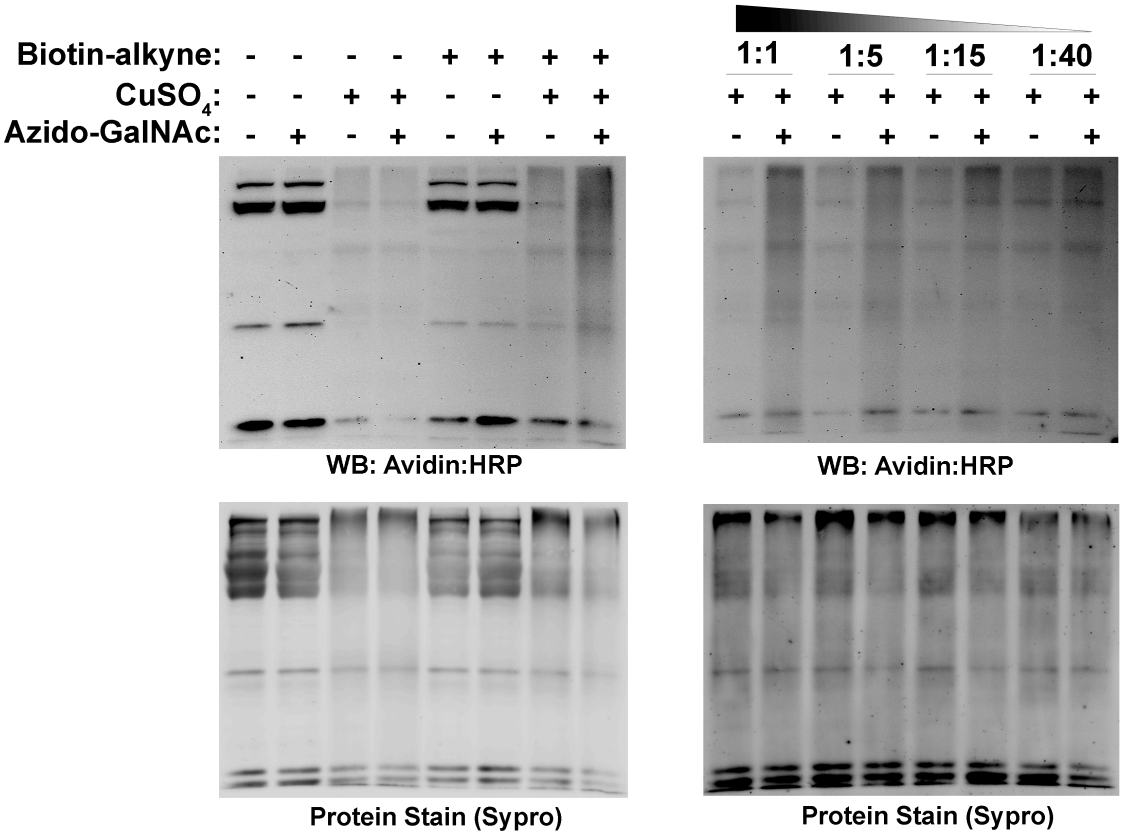

Figure S1: Detection of azido-GalNAc-specific avidin:HRP signal in *C. elegans* cell lysates is dependent on Click Chemistry reaction of lysates with biotin-alkyne and copper catalyst. Primary embryonic N2 *C. elegans* cells were metabolically labeled with GalNAc or azido-GalNAc for 24hrs, then the metabolic label was detected by reacting cell lysates in the described combinations of biotin-alkyne and copper catalyst. Reactions containing a constant amount of copper and various dilutions of biotin-alkyne were also tested.
