# Supplementary material for: Metabolic Labeling of Caenorhabditis elegans Primary Embryonic Cells with Azido-Sugars as a Tool for Glycoprotein Discovery
Source: PLoS One. 2012 Nov 12;7(11):e49020. doi: 10.1371/journal.pone.0049020 (PMC3495777; doi:10.1371/journal.pone.0049020)
Supplement: Figure S2 — A WGA-binding glycosylated isoform of ATP synthase α-subunit is present in C. elegans . C. elegans glycoproteins were solubilized in detergent buffer and purified using WGA-agarose. ATP synthase α-subunit was present in both the non-binding flowthrough (FT) fraction and the fraction specifically eluted with GlcNAc. (PDF) [file pone.0049020.s002.pdf]

Figure S2

Lectin affinity purification (WGA):

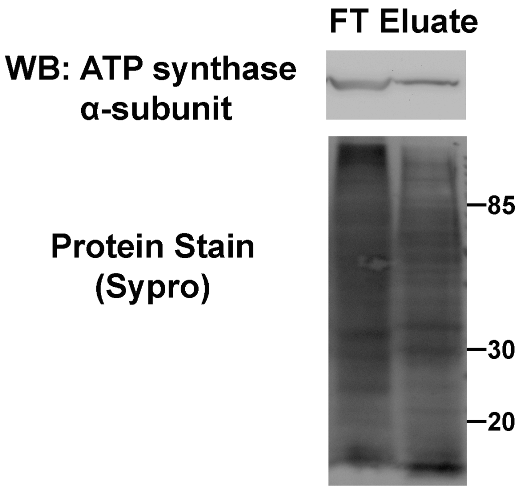

Figure S2: A WGA-binding glycosylated isoform of ATP synthase α-subunit is present in *C. elegans*. *C. elegans* glycoproteins were solubilized in detergent buffer and purified using WGA-agarose. ATP synthase α-subunit was present in both the non-binding flowthrough (FT) fraction and the fraction specifically eluted with GlcNAc.
