# Supplementary material for: Metabolic Labeling of Caenorhabditis elegans Primary Embryonic Cells with Azido-Sugars as a Tool for Glycoprotein Discovery
Source: PLoS One. 2012 Nov 12;7(11):e49020. doi: 10.1371/journal.pone.0049020 (PMC3495777; doi:10.1371/journal.pone.0049020)
Supplement: Figure S3 — The majority of azido-signal from azido-GalNAc treated cells is incorporated into pNGaseF-insensitive glycoproteins. C. elegans ogt-1 cells were incubated with azido-GalNAc, and the lysates were treated with the N-glycan endoglycosidase pNGaseF, mock treated, or left untreated. The remaining azido-labeled glycoproteins were then visualized by Click Chemistry reaction with biotin-alkyne and Western blotting with avidin:HRP. Protein loading was verified by Sypro Ruby total protein staining. The N-glycosylated protein, RNaseB, was run as a positive control for pNGaseF activity. Asterisk indicates the expected molecular weight of pNGaseF. (PDF) [file pone.0049020.s003.pdf]

Figure S3

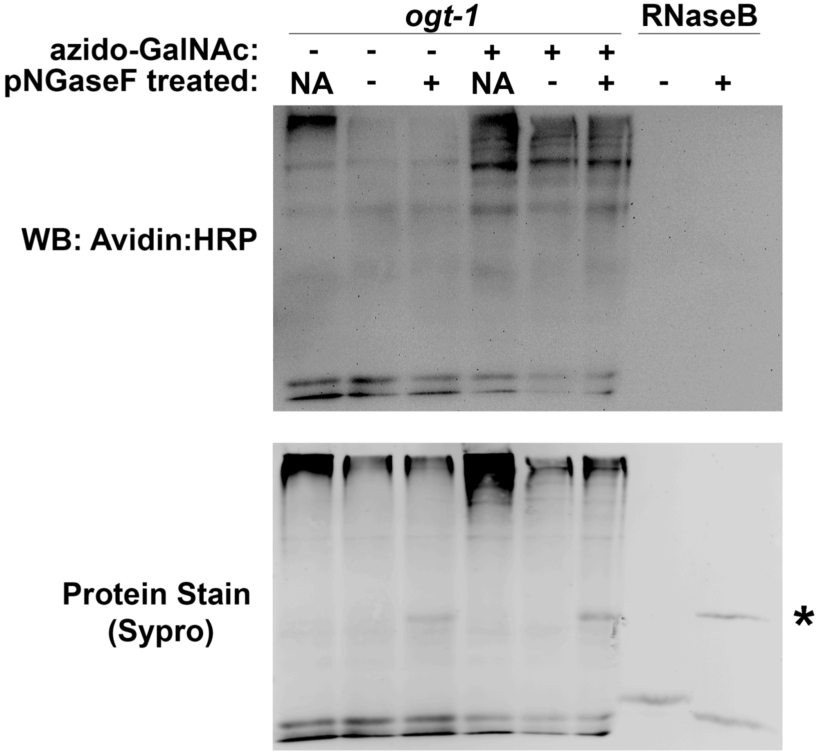

Figure S3: The majority of azido-signal from azido-GalNAc treated cells is incorporated into pNGaseF-insensitive glycoproteins. *C. elegans ogt-1* cells were incubated with azido-GalNAc, and the lysates were treated with the N-glycan endoglycosidase pNGaseF, mock treated, or left untreated. The remaining azido-labeled glycoproteins were then visualized by Click Chemistry reaction with biotin-alkyne and Western blotting with avidin:HRP. Protein loading was verified by Sypro Ruby total protein staining. The N-glycosylated protein, RNaseB, was run as a positive control for pNGaseF activity. Asterisk indicates the expected molecular weight of pNGaseF.
