# Supplementary material for: Metabolic Labeling of Caenorhabditis elegans Primary Embryonic Cells with Azido-Sugars as a Tool for Glycoprotein Discovery
Source: PLoS One. 2012 Nov 12;7(11):e49020. doi: 10.1371/journal.pone.0049020 (PMC3495777; doi:10.1371/journal.pone.0049020)
Supplement: Figure S4 — A portion of C. elegans actin is glycosylated. (A) 450 ug of C. elegans adult lysates were labeled with azido-GalNAc via a mutant β-1, 4-galactosyltransferase (GalT) or mock labeled (no GalT). After reacting the azido-tagged glycoproteins with biotin-alkyne via Click Chemistry, the labeled glycoproteins were purified using streptavidin beads and analyzed by Western blot using an anti-actin antibody (Sigma A4700). Protein loading was visualized by Sypro Ruby total protein staining. Input and flowthrough (FT) lanes contain 20 ug aliquots of each fraction; eluate lane contains the entire fraction. (B) Ten milligrams of C. elegans adult lysates were applied to WGA beads, the beads were washed extensively with PBS, and then the captured proteins were eluted with PBS supplemented with the competing sugar (GlcNAc). Samples were analyzed by Western blot using an anti-actin antibody (Abcam ab3280). Protein loading was visualized by Sypro Ruby total protein staining. Input and FT lanes contain 20 ug aliquots of each fraction; last wash and eluate lanes contain 100% of each fraction. (PDF) [file pone.0049020.s004.pdf]

Figure S4

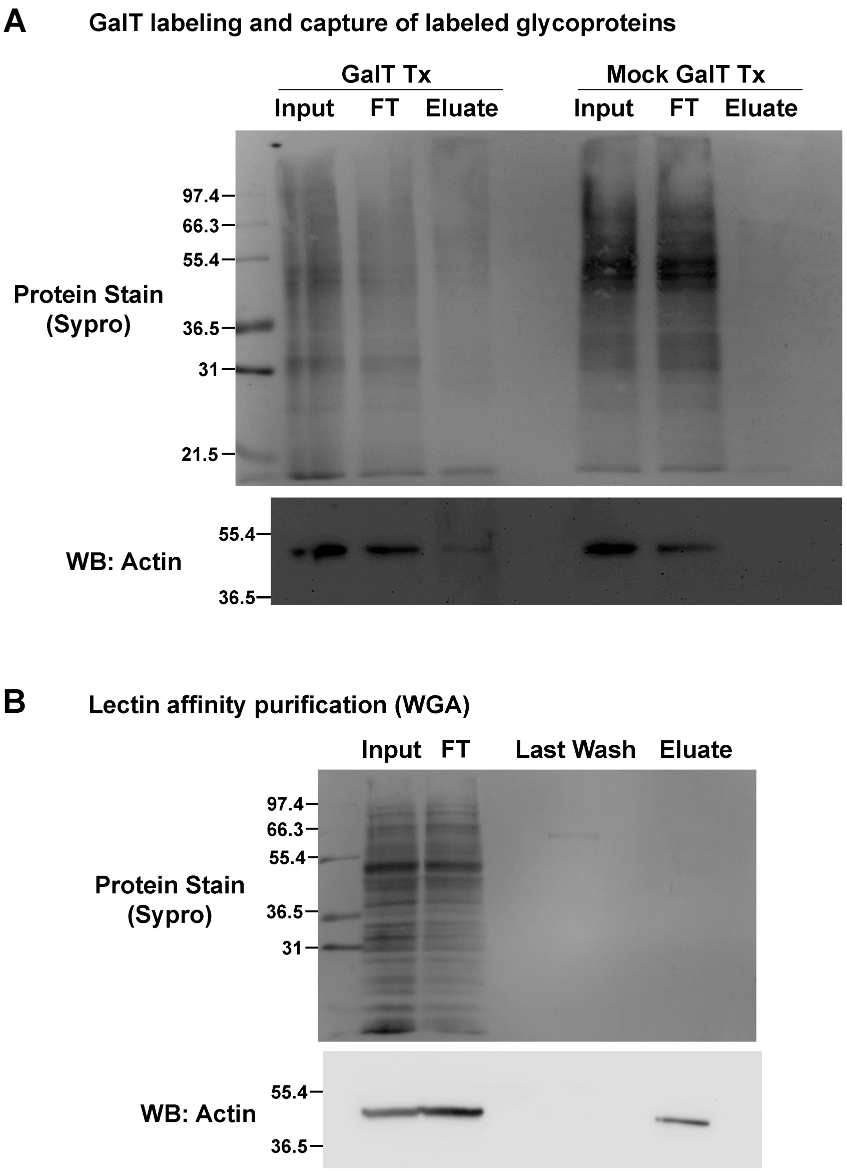

Figure S4: A portion of *C. elegans* actin is glycosylated. (A) 450ug of *C. elegans* adult lysates were labeled with azido-GalNAc via a mutant β-1, 4-galactosyltransferase (GalT) or mock labeled (no GalT). After reacting the azido-tagged glycoproteins with biotin-alkyne via Click Chemistry, the labeled glycoproteins were purified using streptavidin beads and analyzed by Western blot using an anti-actin antibody (Sigma A4700). Protein loading was visualized by Sypro Ruby total protein staining. Input and flowthrough (FT) lanes contain 20ug aliquots of each fraction; eluate lane contains the entire fraction. (B) Ten milligrams of *C. elegans* adult lysates were applied to WGA beads, the beads were washed extensively with PBS, and then the captured proteins were eluted with PBS supplemented with the competing sugar (GlcNAc). Samples were analyzed by Western blot using an anti-actin antibody (Abcam ab3280). Protein loading was visualized by Sypro Ruby total protein staining. Input and FT lanes contain 20ug aliquots of each fraction; last wash and eluate lanes contain 100% of each fraction.
