# Supplementary material for: Metabolic Labeling of Caenorhabditis elegans Primary Embryonic Cells with Azido-Sugars as a Tool for Glycoprotein Discovery
Source: PLoS One. 2012 Nov 12;7(11):e49020. doi: 10.1371/journal.pone.0049020 (PMC3495777; doi:10.1371/journal.pone.0049020)
Supplement: Figure S5 — The azido-labeled protein profile of N2 lysates is comparable to that of ogt-1 lysates. N2 and ogt-1 cells were incubated with 40 µM azido-GalNAc for 72 hrs, then reacted via Click Chemistry with TAMRA-alkyne, fractionated by SDS-PAGE, and electrotransferred. TAMRA signal was detected by UV on the blot, then the blot was stained with Sypro Ruby total protein stain to verify protein loading. (PDF) [file pone.0049020.s005.pdf]

Figure S5

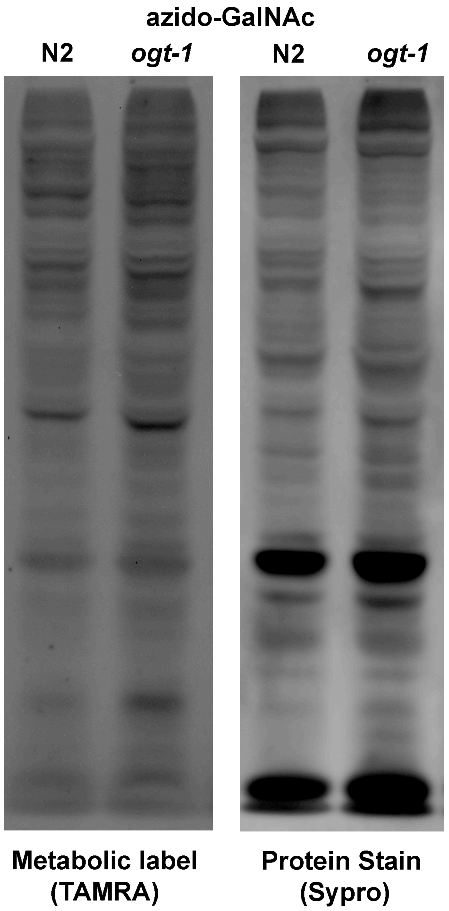

Figure S5: **The azido-labeled protein profile of N2 lysates is comparable to that of *ogt-1* lysates.** N2 and *ogt-1* cells were incubated with 40μM azido-GalNAc for 72hrs, then reacted via Click Chemistry with TAMRA-alkyne, fractionated by SDS-PAGE, and electrotransferred. TAMRA signal was detected by UV on the blot, then the blot was stained with Sypro Ruby total protein stain to verify protein loading.
