# Supplementary material for: Metabolic Labeling of Caenorhabditis elegans Primary Embryonic Cells with Azido-Sugars as a Tool for Glycoprotein Discovery
Source: PLoS One. 2012 Nov 12;7(11):e49020. doi: 10.1371/journal.pone.0049020 (PMC3495777; doi:10.1371/journal.pone.0049020)
Supplement: Figure S6 — ASP-4 is glycosylated. C. elegans glycoproteins were purified using WGA-agarose. The fraction eluted with GlcNAc was fractionated by 2DE and analyzed with Pro-Q Emerald Total Glycoprotein Stain (A) and then with Sypro total protein stain (B). Arrowheads mark the spot identified by mass spectrometry as ASP-4. (PDF) [file pone.0049020.s006.pdf]

# Figure S6

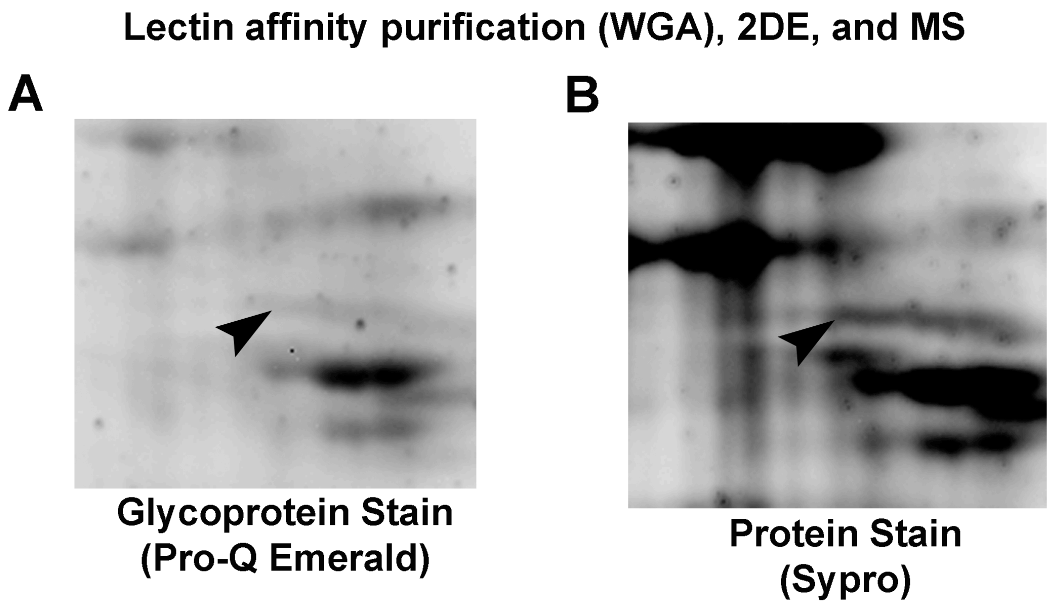


**Figure S6: ASP-4 is glycosylated.** *C. elegans* glycoproteins were purified using WGA-agarose. The fraction eluted with GlcNAc was fractionated by 2DE and analyzed with Pro-Q Emerald Total Glycoprotein Stain (A) and then with Sypro total protein stain (B). Arrowheads mark the spot identified by mass spectrometry as ASP-4.
