# Supplementary material for: Metabolic Labeling of Caenorhabditis elegans Primary Embryonic Cells with Azido-Sugars as a Tool for Glycoprotein Discovery
Source: PLoS One. 2012 Nov 12;7(11):e49020. doi: 10.1371/journal.pone.0049020 (PMC3495777; doi:10.1371/journal.pone.0049020)
Supplement: Supplementary Methods and References S1 — (PDF) [file pone.0049020.s007.pdf]

SUPPLEMENTARY METHODS:

pNGaseF digestion—Samples were reacted for 2hrs at 37˚C with 5µl pNGaseF (New England Biolabs) per every 100µg protein.

*Wheat Germ Agglutinin (WGA) enrichment of C. elegans larvae—S*ynchronized L1/L2 N2 worms were sonicated in PBS then centrifuged at 100,000 x *g* for 30min. The pellet was homogenized in detergent buffer (7M urea, 2M thiourea, 9mM Tris acetate pH 7, 1X protease inhibitor cocktail (CalBiochem), 3% Chaps, 1% Mega 10), and proteins extracted as per (Churchward, M.A., Butt, R.H., et al. 2005). Proteins were incubated with WGA-agarose beads (Vector) for 40 min at RT. Beads were washed 3X 10min in detergent buffer, then proteins eluted with 4X 15min washes of detergent buffer supplemented with 0.25M GlcNAc.

WGA enrichment of C. elegans gravid adults—Synchronized adult N2 worms were boiled for 20min in one volume 1% SDS, 5mM DTT, 20mM HEPES pH 7.9 and 2X protease inhibitor cocktail (CalBiochem) then incubated on ice 10min. Samples were sonicated and then centrifuged 30min at 16,000 x *g*. Ten milligrams of protein lysate from the clarified supernatant were diluted 11-fold into detergent buffer for a final concentration of 1mg/ml and then incubated with WGA-agarose beads for 1hr at RT. Beads were washed 5X 2.5min in 9 bead volumes (BV) PBS. A 6th and final wash of 10min in 9BV PBS was performed, and then captured glycoproteins were eluted by incubating 2 X 10min with 1 BV PBS supplemented with 0.5M GlcNAc. Samples were separated by SDS-PAGE, then transferred to PVDF for Western blot analysis with actin antibody (Abcam ab3280) at 1:3000.

GalT labeling and enrichment—450ug of adult N2 lysates prepared as above were labeled with (or mock-labeled without) mutant GalT and UDP-azido-GalNAc (Life Technologies), as per manufacturer's instructions. Samples were then reacted with biotin-alkyne and subjected to streptavidin-enrichment as described (Nandi, A., Sprung, R., et al. 2006a). Samples were separated by SDS-PAGE, then transferred to PVDF for Western blot analysis with actin antibody (Sigma A4700) at 1:1000.

2D gel electrophoresis and analysis—Precipitated samples were resuspended and isoelectrically focused as per (Schwientek, T., Mandel, U., et al. 2007) with the following modifications: 3-10NL 11cm IPG strips were used; WGA-enriched samples were focused in detergent buffer (see WGA enrichment); 9mg/ml DTT was used for metabolically labeled samples; the final isoelectric focusing step was 8000V for 20,000Vhrs; the first equilibration solution for focused IPG strips contained 2% DTT, the second contained 2.5% iodoacetamide; strips were layered onto 4-15% Criterion Tris HCl gradient gels (Bio-Rad).

WGA–enriched samples were stained with the total glycoprotein stain ProQ Emerald (Invitrogen), imaged with the VersaDoc 4000 (ex 300nm, em 520nm LP), then stained for Sypro Ruby.

Protein identification—The Nevada Proteomics center analyzed selected proteins by trypsin digestion and MALDI TOF/TOF analysis. Spots were digested using a previously described protocol with modifications (Rosenfeld, J., Capdevielle, J., et al. 1992). Samples were washed twice with 25mM ammonium bicarbonate (ABC) followed by 100% acetonitrile, reduced and alkylated using 10 mM dithiothreitol and 100 mM iodoacetamide. Samples were incubated with 75 ng sequencing grade modified porcine trypsin (Promega) in 25 mM ABC for 6 hours at 37C. Samples were spotted onto a MALDI target with a µC18 ZipTip (Millipore Corp, MA). Peptides were eluted off the ZipTip directly onto the target using 5 mg/ml α-cyano-4-hydroxycinnamic acid (in 70% acetonitrile 0.2% formic acid) containing 10 mM ammonium phosphate.

All mass spectrometric data were collected using an ABI 4700 Proteomics Analyzer MALDI TOF/TOF mass spectrometer (Applied Biosystems, CA), using 4000 Series Explorer software v3.6. Peptide masses were acquired in reflectron positive mode (1-keV accelerating voltage) from 650 – 4000 Daltons. Peptide masses were internally calibrated to within 20 ppm using the 842.51 and 2211.10 trypsin autolysis fragments. Spectrum filtering/peak detection settings were S/N threshold 3, cluster area S/N optimization enabled at S/N threshold 10, baseline subtraction enabled at peak width 50. The twenty most intense ions from the MS scan that did not appear on the exclusion list (known trypsin fragments) were subjected to MS/MS. MS/MS settings included: mass range 70 to precursor ion, precursor window relative resolution of 100 FWHM, CID on, metastable suppressor on. Raw spectrum filtering/peak detection settings were S/N threshold 5, cluster area S/N optimization enabled at S/N threshold 6, baseline subtraction enabled at peak width 50.

Peak lists were extracted using ABI's GPS Explorer software v3.6. Analyses were performed as combination MS + MS/MS. MS peak filtering settings: mass range 650 – 4000 Da, minimum S/N 10,

peak density 50 peaks per 200 Da, maximum number of peaks 65. MSMS peak filtering: 50 peaks per 200 Da, Cluster area filter, maximum number of peaks 65. The filtered peak lists were searched using Mascot v 2.2.04 (Matrix Science) using NCBI nr database (NCBI 20090915) containing 8,080,522 sequences. Where stated, NCBI nr database searches were limited to the 27,591 *C. elegans* sequences. Searches were performed without restriction to protein species, Mr, or pI and with variable oxidation of methionine residues and carbamidomethylation of cysteines. Maximum missed cleavage was set to 1 and fragments were limited to trypsin cleavage sites. Precursor mass tolerance and fragment mass tolerance were set to 20ppm and 0.2 Da, respectively when spots passed internal calibration on the MALDI TOF/TOF. Only protein identifications that had significant MASCOT scores (MASCOT scores > 82; MASCOT expect p-value < 0.05) and ≥5 unique peptides were accepted as valid.

References:

Churchward MA, Butt RH, Lang JC, Hsu KK, Coorssen JR. 2005. Enhanced detergent extraction for analysis of membrane proteomes by two-dimensional gel electrophoresis. *Proteome Sci*, 3:5.

Clark PM, Dweck JF, Mason DE, Hart CR, Buck SB, Peters EC, Agnew BJ, Hsieh-Wilson LC. 2008. Direct in-gel fluorescence detection and cellular imaging of O-GlcNAc-modified proteins. *J Am Chem Soc*, 130:11576-11577.

Fan X, She YM, Bagshaw RD, Callahan JW, Schachter H, Mahuran DJ. 2005. Identification of the hydrophobic glycoproteins of Caenorhabditis elegans. *Glycobiology*, 15:952-964.

Hu Y, Suarez J, Fricovsky E, Wang H, Scott BT, Trauger SA, Han W, Oyeleye MO, Dillmann WH. 2009. Increased enzymatic O-GlcNAcylation of mitochondrial proteins impairs mitochondrial function in cardiac myocytes exposed to high glucose. *J Biol Chem*, 284:547-555.

Kung LA, Tao SC, Qian J, Smith MG, Snyder M, Zhu H. 2009. Global analysis of the glycoproteome in Saccharomyces cerevisiae reveals new roles for protein glycosylation in eukaryotes. *Mol Syst Biol*, 5:308.

Nandi A, Sprung R, Barma DK, Zhao Y, Kim SC, Falck JR. 2006a. Global identification of O-GlcNAc-modified proteins. *Anal Chem*, 78:452-458.

Nandi A, Sprung R, Barma DK, Zhao Y, Kim SC, Falck JR, Zhao Y. 2006b. Global identification of O-GlcNAc-modified proteins. *Anal Chem*, 78:452-458.

Rosenfeld J, Capdevielle J, Guillemot JC, Ferrara P. 1992. In-gel digestion of proteins for internal sequence analysis after one- or two-dimensional gel electrophoresis. *Anal Biochem*, 203:173-179.

Schwientek T, Mandel U, Roth U, Muller S, Hanisch FG. 2007. A serial lectin approach to the mucin-type O-glycoproteome of Drosophila melanogaster S2 cells. *Proteomics*, 7:3264-3277.

Sprung R, Nandi A, Chen Y, Kim SC, Barma D, Falck JR, Zhao Y. 2005. Tagging-via-substrate strategy for probing O-GlcNAc modified proteins. *J Proteome Res*, 4:950-957.

Teo CF, Ingale S, Wolfert MA, Elsayed GA, Not LG, Chatham JC, Wells L, Boons GJ. 2010. Glycopeptide-specific monoclonal antibodies suggest new roles for O-GlcNAc. *Nat Chem Biol*, 6:338-343.
