# Supplementary material for: Metabolic Labeling of Caenorhabditis elegans Primary Embryonic Cells with Azido-Sugars as a Tool for Glycoprotein Discovery
Source: PLoS One. 2012 Nov 12;7(11):e49020. doi: 10.1371/journal.pone.0049020 (PMC3495777; doi:10.1371/journal.pone.0049020)
Supplement: Table S1 — C. elegans glycoproteins candidates detected by metabolic labeling of primary embryonic cells with azido-GalNAc. C. elegans cells were labeled with azido-GalNAc, the lysates reacted with TAMRA-alkyne via Click Chemistry, and the proteins separated by 2DE. The 2DE glycoprotein candidate spots selected for MALDI-TOF/TOF identification were those that in two independent experiments exhibited TAMRA fluorescence only in the azido-GalNAc gel or >3-fold higher TAMRA fluorescence in the azido-GalNAc gel vs. the GalNAc gel. A protein identification was considered valid only if it was supported by a significant MASCOT score (MASCOT score >82; MASCOT expect p-value <0.05) and more than 5 unique peptides. For those 2DE spots with valid protein identifications, the top-scoring MASCOT identification from a search of the NCBI nr database is listed for each 2DE spot. (PDF) [file pone.0049020.s008.pdf]

Table S1. *C. elegans* glycoproteins candidates were detected by metabolic labeling of primary embryonic cells with azido-GalNAc.

Protein ID	NCBI protein accession number	% coverage	Number of unique peptides	Mascot score	Mascot expect value	Number of matched peaks	Number of unmatched peaks	Ortholog reported as glycoprotein	Mitochondrially -annotated
hypothetical protein T08B2.7	gi|17508953	48	34	567	1.60E-50	44	44		Yes
CYclophyliN family member (cyn-7)	gi|17566266	53<br>44	9<br>6	342<br>120	5.10E-28<br>8.10E-06	15<br>11	47<br>44	E	
Stress Induced Protein family member (sip-1)	gi|17554792	74	9	411	6.40E-35	16	43		
Ubiquinol-Cytochrome c oxidoReductase complex family member (ucr-2.3)	gi|17555260	37	11	485	2.60E-42	19	80		Yes
Acyl CoA DeHydrogenase family member (acdh-12)	gi|71985184	50	34	438	1.30E-37	46	61		Yes
VITellogenin structural genes family member (vit-6)	gi|71991083	23	32	314	3.20E-25	45	52	G (Reported in *C. elegans*)	
hypothetical protein C37E2.1	gi|17550822	42	17	378	1.30E-31	26	32	A	Yes
TU elongation Factor (EF-Tu), Mitochondrial family member (tufm-1)	gi|17556456	42	24	483	4.0E-42	35	37	A	Yes
hypothetical protein ZK829.4	gi|17544676	48<br>48<br>47<br>44	24<br>26<br>27<br>24	369<br>592<br>746<br>360	1.00E-30<br>5.10E-53<br>2.00E-68<br>8.10E-30	31<br>40<br>41<br>34	89<br>61<br>42<br>58		Yes
hypothetical protein Y37E3.17	gi|71994045	19	14	193	4.00E-13	23	22		Yes

PeRoxireDoXin family member (prdx-2)	gi|32565831	46	8	282	5.10E-22	12	100	A, F	
* Tubulin, Beta family member (tbb-1)	gi|17553980	40<br>24	19<br>11	499<br>121	1.00E-43<br>6.40E-06	30<br>18	71<br>22	A	
Small Glutamine-rich Tetratrico repeat protein family member (sgt-1)	gi|17535447	23	6	167	1.60E-10	12	21	F	
ACTin family member (act-4)	gi|71994099	44	15	399	1.00E-33	20	75	A	
hypothetical protein C04C3.3	gi|17538422	34	9	344	3.20E-28	16	41	B	Yes
H28O16.1 (phi-37)	gi|71988080	43	22	405	2.60E-34	34	54	C	Yes
ASpartyl Protease family member (asp-4)	gi|17549909	14	6	125	2.60E-06	10	110		
ALDOlase (fructose bisphosphate aldolase) (T05D4.1)	gi|25150450	60	17	305	8.70E-27	25	95	A, D	
Elongation FacTor family member(eft-2)	gi|17506493	14	12	163	1.40E-12	17	103	A	
TCTP (translationally-controlled tumor protein) homolog family member (tct-1)	gi|17506817	72	11	500	2.80E-46	18	102	C	

C. elegans cells were labeled with azido-GalNAc, the lysates reacted with TAMRA-alkyne via Click Chemistry, and the proteins separated by 2DE. The 2DE glycoprotein candidate spots selected for MALDI-TOF/TOF identification were those that in two independent experiments exhibited TAMRA fluorescence only in the azido-GalNAc gel or >3-fold higher TAMRA fluorescence in the azido-GalNAc gel *vs.* the GalNAc gel. A protein identification was considered valid only if it was supported by a significant MASCOT score (MASCOT score > 82; MASCOT expect p-value < 0.05) and more than 5 unique peptides. For those 2DE spots with valid protein identifications, the top-scoring MASCOT identification from a search of the NCBI nr database is listed for each 2DE spot. *For these spots, the top-scoring identification from a search of the NCBI nr database was beta tubulin isoform 1 but from a species other

than *C. elegans*; thus the data from the top-scoring identification from a search of the *C. elegans* database is listed instead. Mitochondrial annotation was determined from the Wormbase.org and Uniprot databases. A; Nandi et al. (Nandi, A., Sprung, R., et al. 2006); B: Clark et al. (Clark, P.M., Dweck, J.F., et al. 2008); C: Kung et al. (Kung, L.A., Tao, S.C., et al. 2009); D: Hu et al. (Hu, Y., Suarez, J., et al. 2009); E: Teo et al. (Teo, C.F., Ingale, S., et al. 2010); F: Sprung et al. (Sprung, R., Nandi, A., et al. 2005); G: Fan et al (Fan, X., She, Y.M., et al. 2005)
