# Supplementary material for: Metabolic Labeling of Caenorhabditis elegans Primary Embryonic Cells with Azido-Sugars as a Tool for Glycoprotein Discovery
Source: PLoS One. 2012 Nov 12;7(11):e49020. doi: 10.1371/journal.pone.0049020 (PMC3495777; doi:10.1371/journal.pone.0049020)
Supplement: Table S2 — MALDI-TOF/TOF identifications of candidate glycoproteins from 2DE protein spots or SDS-PAGE gel bands. WGA affinity purification: C. elegans larval proteins were captured with WGA-agarose, eluted with GlcNAc, and separated by 2DE. A spot that fluoresced with Pro-Q Emerald Total Glycoprotein Stain of the appropriate molecular weight and isoelectric point to be ASP-4 was selected for MALDI-TOF/TOF identification. GalT labeling: C. elegans L1/L2 lysates treated or mock treated with GalT and UDP-azido-GalNAc were reacted with biotin-alkyne. Biotinylated azido-glycoproteins were then captured with avidin beads. One SDS-PAGE protein band captured specifically from GalT-treated lysates and not mock-treated lysates was selected for MALDI-TOF/TOF identification. The top-scoring MASCOT identification from a search of the NCBI nr database or, where stated, the C. elegans database, is listed for each 2DE spot or SDS-PAGE gel band. (PDF) [file pone.0049020.s009.pdf]

**Table S2: MALDI-TOF/TOF identifications of candidate glycoproteins from 2DE protein spots or SDS-PAGE gel bands.**

| Experiment | Protein ID | NCBI protein accession number | % coverage | Number of unique peptides | Mascot score | Mascot expect value | Number of matched peaks | Number of unmatched peaks |
|---|---|---|---|---|---|---|---|---|
| WGA affinity purification | ASP-4 (*C. elegans*) | gi\|17549909 | 19 | 8 | 263 | 4.00E-20 | 14 | 106 |
| GalT labeling | Actin beta (*Oncorhynchus mykiss*) | gi\|185132289 | 38 | 14 | 420 | 8.10E-36 | 23 | 109 |
| | Searched *C. elegans* database: ACT-4 (*C. elegans*) | gi\|71994099 | 34 | 10 | 295 | 8.70E-26 | 19 | 114 |

WGA affinity purification: *C. elegans* larval proteins were captured with WGA-agarose, eluted with GlcNAc, and separated by 2DE. A spot that fluoresced with Pro-Q Emerald Total Glycoprotein Stain of the appropriate molecular weight and pI to be ASP-4 was selected for MALDI-TOF/TOF identification. GalT labeling: *C. elegans* L1/L2 lysates treated or mock treated with GalT and UDP-azido-GalNAc were reacted with biotin-alkyne. Biotinylated azido-glycoproteins were then captured with avidin beads. One SDS-PAGE protein band captured specifically from GalT-treated lysates and not mock-treated lysates was selected for MALDI-TOF/TOF identification. The top-scoring MASCOT identification from a search of the NCBI nr database or, where stated, the *C. elegans* database, is listed for each 2DE spot or SDS-PAGE gel band.
