# Supplementary material for: Metabolic Labeling of Caenorhabditis elegans Primary Embryonic Cells with Azido-Sugars as a Tool for Glycoprotein Discovery
Source: PLoS One. 2012 Nov 12;7(11):e49020. doi: 10.1371/journal.pone.0049020 (PMC3495777; doi:10.1371/journal.pone.0049020)
Supplement: Dataset S2 — Mass spectrometry data from glycoprotein verification experiments supporting the identification of C. elegans ASP-4 and actin. ASP-4: C. elegans glycoproteins were purified using WGA-agarose. The fraction eluted with GlcNAc was fractionated by 2DE. Protein spots that stained positive with Pro-Q Emerald Total Glycoprotein Stain were analyzed by MALDI-TOF/TOF, and the mass spectrometry data pertaining to the spot identified as ASP-4 is shown. Actin: Glycoproteins containing terminal GlcNAc were purified by treating N2 larval lysates with both UDP-azido-GalNAc and GalT (+), or with UDP-azido-GalNAc alone (−),reacting with biotin-alkyne, and capturing the biotinylated glycoproteins with streptavidin beads. Eluted glycoproteins were fractionated by SDS-PAGE, and the gel was stained with Sypro. A protein band unique to the GalT treated sample was identified by MALDI-TOF/TOF as ACT-4; although the peptides observed are shared by C. elegans ACT-2, ACT-3, and ACT-4. Dataset S2 includes the following information in support of the identification of ASP-4 and actin: protein accession number, MASCOT score and expect value, sequence coverage, peptides observed, ion score if MS/MS was performed on the peptide, and the MS spectra (m/z vs. intensity). (PDF) [file pone.0049020.s011.pdf]

Match to: **gi|17549909** Score: **263** Expect: **4e-020**

ASpartyl Protease family member (asp-4) [Caenorhabditis elegans]

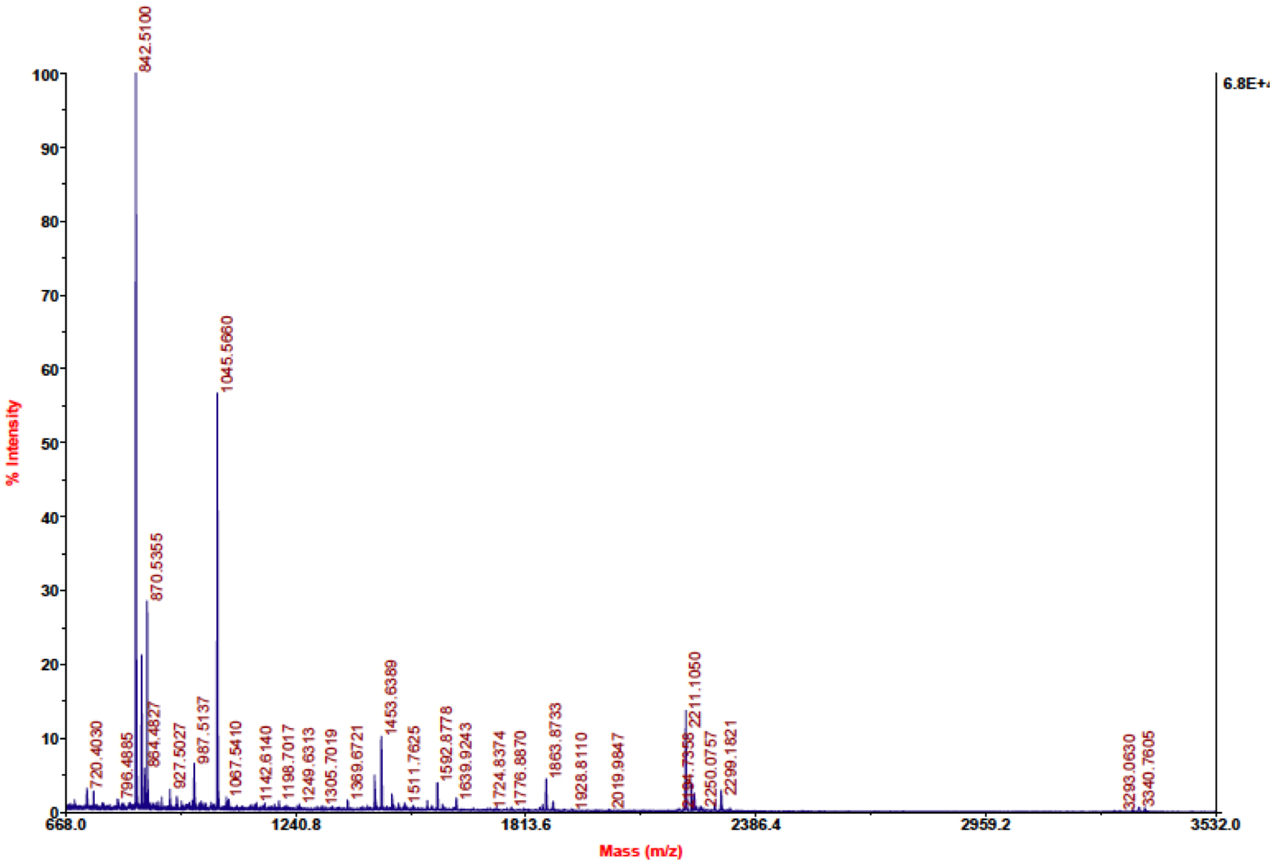

Start - End	Observed	Mr(expt)	Mr(calc)	ppm	Miss	Sequence	
228 - 239	1479.7979	1478.7906	1478.7670	16	0	K.VPSNLFSFWLNR.N	
228 - 239	1479.7979	1478.7906	1478.7670	16	0	K.VPSNLFSFWLNR.N	
240 - 257	1863.8732	1862.8659	1862.8646	1	0	R.NPDSEIGGEITFGGIDSR.R	(Ions score 94)
240 - 257	1863.8732	1862.8659	1862.8646	1	0	R.NPDSEIGGEITFGGIDSR.R	
258 - 270	1592.8778	1591.8705	1591.8722	-1	1	R.RYVEPITYVPVTR.K	(Ions score 21)
258 - 270	1592.8778	1591.8705	1591.8722	-1	1	R.RYVEPITYVPVTR.K	
259 - 270	1436.7776	1435.7703	1435.7711	-1	0	R.YVEPITYVPVTR.K	(Ions score 31)
259 - 270	1436.7776	1435.7703	1435.7711	-1	0	R.YVEPITYVPVTR.K	
272 - 280	1218.5554	1217.5481	1217.5539	-5	1	K.GYWQFKMDK.V	Oxidation (M)
309 - 325	1855.0264	1854.0191	1854.0251	-3	0	K.AQIEAIQNFIGAEPLIK.G	
396 - 406	1453.6389	1452.6316	1452.6310	0	0	R.YYSVFDFDQNR.V	(Ions score 71)
396 - 406	1453.6389	1452.6316	1452.6310	0	0	R.YYSVFDFDQNR.V	
407 - 413	720.4031	719.3959	719.3966	-1	0	R.VGFAQAK.T	(Ions score 18)
407 - 413	720.4031	719.3959	719.3966	-1	0	R.VGFAQAK.T	

Match to: **gi|71994099** Score: **295** Expect: **8.7e-026**

ACTin family member (act-4) [Caenorhabditis elegans]

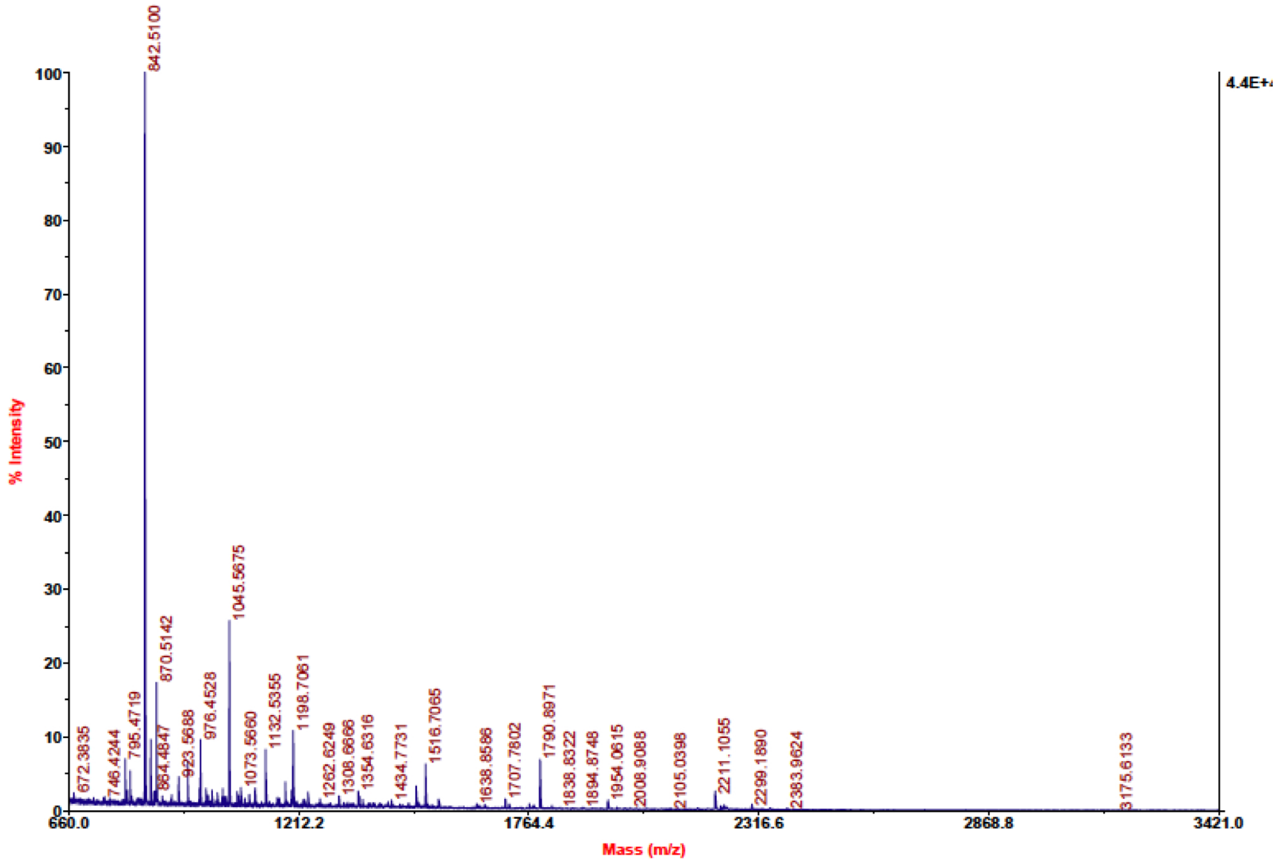

Start - End	Observed	Mr(expt)	Mr(calc)	ppm	Miss	Sequence	
6 - 15	976.4528	975.4455	975.4410	5	0	R.AGFAGDDAPR.A	(Ions score 34)
6 - 15	976.4528	975.4455	975.4410	5	0	R.AGFAGDDAPR.A	
16 - 26	1198.7059	1197.6986	1197.6982	0	0	R.AVFPSIVGRPR.H	(Ions score 23)
16 - 26	1198.7059	1197.6986	1197.6982	0	0	R.AVFPSIVGRPR.H	
38 - 49	1354.6316	1353.6243	1353.6161	6	1	K.DSYVGDEAQSKR.G	(Ions score 31)
38 - 49	1354.6316	1353.6243	1353.6161	6	1	K.DSYVGDEAQSKR.G	
49 - 55	800.5206	799.5134	799.5280	-18	1	K.RGILTLK.Y	
83 - 100	1954.0614	1953.0541	1953.0571	-2	0	R.VAPEEHPVLLTEAPLNPK.A	
184 - 193	1132.5358	1131.5285	1131.5197	8	0	R.GYSFTTTAER.E	(Ions score 41)
184 - 193	1132.5358	1131.5285	1131.5197	8	0	R.GYSFTTTAER.E	
226 - 241	1776.8818	1775.8745	1775.8690	3	0	K.SYELPDGQVITVGNER.F	
279 - 299	2231.0725	2230.0652	2230.0576	3	0	K.DLYANTVLSGGTTMYPGIADR.M	Oxidation (M)
316 - 322	795.4719	794.4646	794.4650	-0	0	K.IIAPPER.K	(Ions score 18)
316 - 322	795.4719	794.4646	794.4650	-0	0	K.IIAPPER.K	
316 - 323	923.5689	922.5616	922.5600	2	1	K.IIAPPERK.Y	(Ions score 2)
316 - 323	923.5689	922.5616	922.5600	2	1	K.IIAPPERK.Y	
347 - 359	1516.7065	1515.6992	1515.6954	3	0	K.QEYDESGPSIVHR.K	(Ions score 85)
347 - 359	1516.7065	1515.6992	1515.6954	3	0	K.QEYDESGPSIVHR.K	
347 - 360	1644.8157	1643.8084	1643.7903	11	1	K.QEYDESGPSIVHRK.C	
